# UAV-based individual plant detection and geometric parameter extraction in vineyards

**DOI:** 10.3389/fpls.2023.1244384

**Published:** 2023-11-14

**Authors:** Meltem Cantürk, Laura Zabawa, Diana Pavlic, Ansgar Dreier, Lasse Klingbeil, Heiner Kuhlmann

**Affiliations:** Institute of Geodesy and Geoinformation, University of Bonn, Bonn, Germany

**Keywords:** precision viticulture, grapevine detection, vineyard canopy characteristics, 3D vineyard structure, UAV-based point cloud

## Abstract

Accurately characterizing vineyard parameters is crucial for precise vineyard management and breeding purposes. Various macroscopic vineyard parameters are required to make informed management decisions, such as pesticide application, defoliation strategies, and determining optimal sugar content in each berry by assessing biomass. In this paper, we present a novel approach that utilizes point cloud data to detect trunk positions and extract macroscopic vineyard characteristics, including plant height, canopy width, and canopy volume. Our approach relies solely on geometric features and is compatible with different training systems and data collected using various 3D sensors. To evaluate the effectiveness and robustness of our proposed approach, we conducted extensive experiments on multiple grapevine rows trained in two different systems. Our method provides more comprehensive canopy characteristics than traditional manual measurements, which are not representative throughout the row. The experimental results demonstrate the accuracy and efficiency of our method in extracting vital macroscopic vineyard characteristics, providing valuable insights for yield monitoring, grape quality optimization, and strategic interventions to enhance vineyard productivity and sustainability.

## 1 Introduction

Enhancing and optimizing the productivity and quality of grapevine crops is a primary goal for winegrowers, making vineyard management decisions significant (Moreno and Andújar, 2023). A key factor in achieving this lies in obtaining precise and detailed information about the overall structure of vineyards, which encompasses plant arrangements and geometric canopy attributes. This information plays a pivotal role in making well-informed decisions that are essential for tasks like pruning, applying pesticides, and maximizing yield (De Castro et al., 2018). Plant-wise canopy characteristics offer insights into plant vigor, which is crucial for informed decisions during the growth season. Despite the challenge of measuring these attributes across the entire vineyard, such as estimating per-plant volumes, their inspection can provide valuable information. This, in turn, has the potential to significantly impact the precise application of sprayed substances, enhancing overall vineyard management strategies (Caruso et al., 2017).

Key geometric parameters of grapevine crops, such as canopy structure, height, width, volume, and leaf area, are closely connected to plant growth, health, and potential yield. These factors allow breeders to identify and efficiently manage distinct vineyard areas, optimizing their cultivation strategies (Moreno and Andújar, 2023). Estimation of these parameters is traditionally performed by human operators collecting manual measurements about the canopy characteristics. However, as this task is labor intensive, these parameters are typically extrapolated from a small sub-section of the vineyard, preventing farmers from making optimal decisions at the individual plant level (Zabawa et al., 2020). Thus, automating the identification and accurate mapping of individual vine rows and trunks becomes crucial for precisely evaluating the vineyard’s state (Jurado et al., 2020; Biglia et al., 2022). Recently, Unmanned Aerial Vehicles (UAVs) have been commonly used for this task due to their efficient data acquisition, simplicity, and cost-effectiveness (Matese and Di Gennaro, 2015). UAVs can quickly cover large vineyard areas and capture high-resolution images at low altitudes, offering advantages over ground-based, satellite, and aircraft systems (Ferro and Catania, 2023).

Although some grapevine parameters can be extracted from single images, a complete 3D vineyard model is more effective for investigating conditions under the canopy and deriving traits like biomass, canopy volume, and vine-row width and height (Weiss and Baret, 2017; Pádua et al., 2018; Pádua et al., 2019; Tsouros et al., 2019; Di Gennaro and Matese, 2020; Ferro and Catania, 2023). For an accurate 3D vineyard model, different sensor modalities can be used, including LiDAR sensors, Terrestrial Laser Scanners (TLS), and RGB cameras combined with structure from motion (SfM) algorithm (Remondino and El-Hakim, 2006). LiDAR and SfM point clouds have distinct characteristics that impact their suitability for vineyard plant phenotyping, and several studies compared the accuracy of the two point clouds. In one study (Madec et al., 2017), both UAV-based SfM and ground-based LiDAR showed comparable accuracy in wheat crop height determination, while another (Petrović et al., 2022) showed SfM point cloud superior accuracy in representing grapevine canopies due to its higher data density capture based on ground sampling distance. The affordability of RGB cameras compared to LiDAR has sparked interest, leading to numerous studies utilizing SfM-derived point clouds to estimate vineyard parameters (De Castro et al., 2018; Matese and Di Gennaro, 2018; Jurado et al., 2020; Pádua et al., 2020).

Accurately determining the location of individual plants within a vineyard is crucial for precision vineyard management tasks like selective harvesting, accurate spraying, fertilization and weeding, and effective crop management (Milella et al., 2019). In a related study, Milella et al. (2019) proposed an algorithm using an affordable RGB-D sensor on an agricultural vehicle to estimate per-plant canopy volume via k-means clustering of a reconstructed 3D vine row. However, this method requires knowing the exact plant count (k) and spacing, which is unfeasible for larger vineyards where the number of plants and their spacing can vary significantly between vineyards. Additionally, they segmented images into grape bunches, leaves, and trunks but they did not explore individual trunk detection and only tested on a single vine row. In another study, Jurado et al. (2020) described an automatic method for identifying and locating individual grapevine trunks, posts, and missing plants based on spatial segmentation without using prior knowledge of the number of plants and the distance between plants. However, this method cannot provide the canopy parameters of the vineyard but just the individual plants’ locations within a point cloud. Both of these research efforts highlight the challenges of accurately estimating vineyard parameters, particularly when dealing with large-scale vineyards. While they contribute valuable methods for plant detection and identification, they each have limitations regarding the information they can provide about the vineyard as a whole.

Several studies investigated the estimation of geometric canopy characteristics. Mathews and Jensen (2013) utilized the SfM technique to construct a 3D vineyard point cloud to estimate the vine leaf area index (LAI). Furthermore, Weiss and Baret (2017) developed an algorithm that utilizes dense point clouds derived from an SfM algorithm to estimate crucial vineyard structural attributes like row orientation, height, width, and spacing. Similarly, Comba et al. (2018) introduced an unsupervised algorithm for vineyard detection and evaluation of vine-row attributes such as vine rows orientation and inter-rows spacing based on the 3D point cloud. Subsequently, Comba et al. (2019) extended the utilization of 3D point clouds by integrating multispectral and thermal images with RGB data to perform a comprehensive characterization of vineyard vigor. Mesas-Carrascosa et al. (2020) classified 3D point cloud into vegetation and soil using RGB information through color vegetation indices (CVIs) and calculated the height of vines with respect to the classified soil. In a distinct approach, Di Gennaro and Matese (2020) implemented the 2.5D-surface and 3D-alpha shape approaches to build an unsupervised and integrated procedure for biomass estimation and missing plant detection in a vineyard. All the above approaches estimate a subset of the necessary parameters for vineyard management. To the best of our knowledge, no single automatic pipeline capable of concurrently estimating a large set of vine canopy traits from 3D point clouds has been proposed in the literature.

The contribution of this paper is a pipeline to determine single plant locations in a vineyard from UAV-derived point clouds. Additionally, we extract geometric parameters like plant height, width, and volume along the row with a high spatial resolution, making it possible to assign the values to the detected single plants. We demonstrate the method’s capability with several datasets generated with an SfM approach using UAV imagery. We also analyze which flight parameters are suitable for the task. Finally, we show the results derived from UAV-based LiDAR data without changing any parameters of the pipeline. This demonstrates that our pipeline significantly reduces the need for manual parameter tuning and can be successfully applied to different 3D sensors.

## 2 Materials and methods

### 2.1 Study site and data acquisition

**Figure 1:**
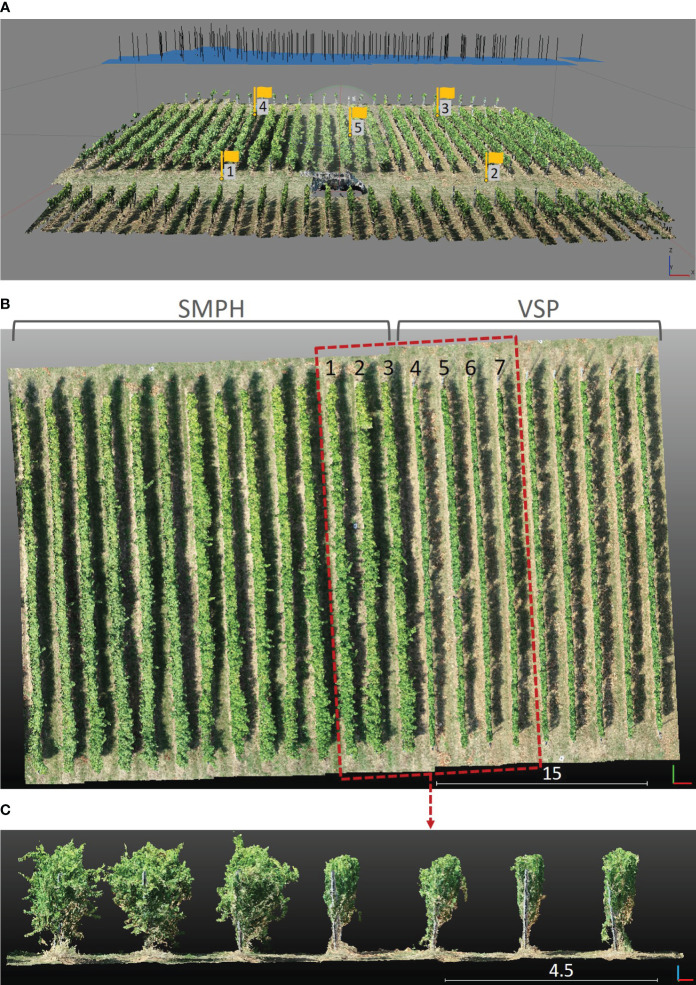
**(A)** The camera positions at the moment of image acquisition and reconstructed 3D point cloud. The 5 yellow flags represent the GCPs. **(B)** Top view of the reconstructed 3D point cloud. The area in the red rectangle was selected as a subset. **(C)** Side view of the subset that comprises 3 rows of the SMPH training system (left) and 4 rows of the VSP training system (right).

The study area is located in the experimental vineyard plots of JKI Geilweilerhof in Siebeldingen, Germany. The institute aims to breed new cultivars resistant to grapevine disease, weather-related stress factors, and high-quality wine production. The vineyard plot was composed of 23 rows, comprising 14 rows trained in the semi-minimal pruned hedge (SMPH) system and 9 rows in the vertical shoot positioning (VSP) system, as illustrated in **Figure 1**. Our investigation focused on assessing the accuracy of the proposed method within two distinct training systems, both characterized by irregular vine spacing.

The VSP system has been commonly used in traditional grape cultivation in Germany due to its suitability for cool climates. However, this system requires labor-intensive tasks like winter pruning and wire positioning, leading to high labor costs. To address this challenge and reduce manual labor expenses, a new training method called SMPH was introduced. SMPH aims to optimize grapevine growth and canopy development while minimizing the need for time-consuming pruning and maintenance activities (Kraus et al., 2018). The SMPH pruning system for grapevines results in notable physiological and morphological changes compared to the traditional VSP trellis. VSP has a single main branch that grows over several years, and the rest gets removed after each growth period. Every year, the other branches regrow. The grapes are mostly positioned near the bottom of the canopy where they are rarely occluded (Zabawa et al., 2019). On the other hand, SMPH allows branches to remain in the trellis after the growing season, creating a more voluminous canopy with older wood and smaller leaves that bud earlier in spring than the VSP. Moreover, it changes the grape cluster structure, producing smaller berries on longer stalks in looser clusters (Pennington, 2019). Two different training systems can be seen in detail in **Figure 1C**. This distinction highlights the variety of cultivation practices employed within the vineyard, enriching the scope of our study.

This study used two different setups for data collection and generating the vineyard point cloud. The first setting is based on a DJI Phantom 4 Pro quadcopter UAV, equipped with an onboard RGB camera. Three flights were conducted over one plot (49°13’10.4” N, 8°02’33.5” E) with different heights and camera angles. The flight parameters of the UAV measurements can be seen in **Table 1**. To obtain the absolute coordinates of the 3D point cloud, 5 ground control points (GCPs) have been measured using the Leica GS18 GNSS RTK system, with 2-3 cm accuracy in position and height. The camera positions at the time of image acquisition at 15 m height and with a nadir angle can be seen in **Figure 1A**. The point cloud was reconstructed using images with a combination of three different flight parameters and individually with the images belonging to each flight number. This first study setting utilized the SfM technique in Agisoft Metashape Professional (version 1.7.4) to generate 3D point clouds. Aerial images acquired with three different flight parameter settings are aligned using the software automatically identifying features from each image (Che et al., 2020; Wu et al., 2022). For each flight, GCPs were used to get a georeferenced dense point cloud. Since the UAV was equipped with an RGB camera, the result was a 3D point cloud including RGB information. The area in the red rectangle in **Figure 1B** was manually selected as a subset to investigate the impact of different flight parameters on the extracted plant parameters. The subset included three rows (1, 2, and 3) trained in SMPH, and four rows (4, 5, 6, and 7) trained in VSP. As a result, we obtained four different point clouds, one of them being the *combined* dataset, while the others were associated with their respective flight parameters: *tilted_20m*, *nadir_20m*, and *nadir_15m*.

**Table 1:** Flight parameters of the UAV measurements.

	*tilted_20m*	*nadir_20m*	*nadir_15m*
Flight height	20 m	20 m	15 m
Camera angle	65°	Nadir	Nadir
Number of images	88	76	153

The second experimental setup for this study is based on a DJI Matrice 600 Pro UAV equipped with a Riegl miniVUX-2UAV laser scanner with a 200kHz pulse repetition rate and a 15 mm accuracy at 50 m distance. Furthermore, the platform has pose estimation sensors onboard, including the Inertial Measurement Unit (IMU) Applanix APX-20 and GNSS antenna Applanix AV14. For this second setting, we used another vineyard plot (49°13’03” N, 8°02’49.5” E). The resulting georeferenced LiDAR point cloud had horizontal accuracy below 0.05 m and vertical accuracy under 0.10 m.

### 2.2 Point cloud processing pipeline

**Figure 2:**
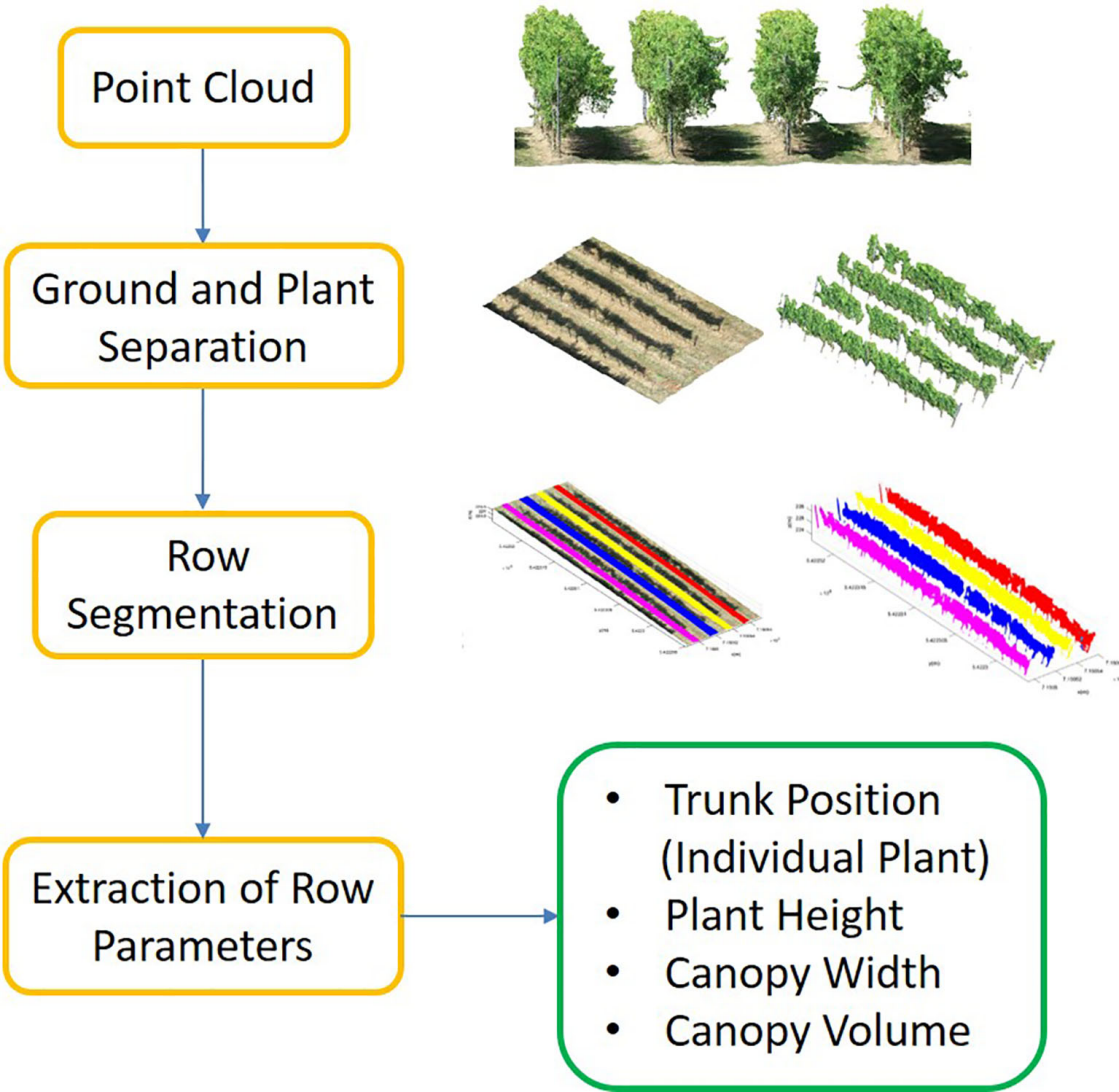
Flowchart of the general framework for row parameter extraction.

We proposed a pipeline to extract different phenotypic traits of the vineyard such as plant height, canopy width, and canopy volume as well as individual plant (trunk) positions. The pipeline included three main steps; (i) point cloud separation into the ground (*GroundPCl*) and plant (*PlantPCl*), (ii) individual row segmentation, and (iii) extraction of row parameters. The study workflow is shown in **Figure 2**. Furthermore, the study utilized the software Matlab 2022b for point cloud processing and CloudCompare 2.12 for point cloud visualization.

#### 2.2.1 Ground-plant separation and height normalization

**Figure 3:**
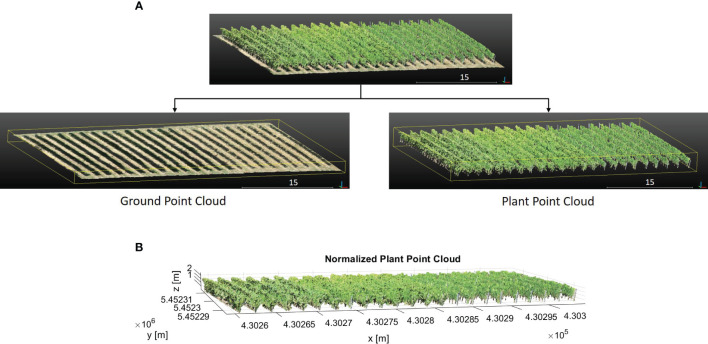
**(A)** The segmentation of the whole point cloud into plant and ground. **(B)** The height-normalized *PlantPCl*.

To extract plant parameters, the point cloud was separated into ground and plant using the Cloth Simulation Filtering (CSF) algorithm (Zhang et al., 2016) in CloudCompare and defined as *Ground PCl* and *Plant PCl*. The CSF algorithm is well-suited for rugged and sloping terrains (Liu et al., 2021). Since the vineyard was located in a sloped area, we used the CSF algorithm to separate ground and plant. The algorithm inverts the point cloud and then covers the inverted surface with a simulated cloth. Using the interactions between the cloth nodes and the corresponding points, the point cloud can be separated into the ground and non-ground points. The main two parameters we tuned in this algorithm are grid resolution *GR* and distance threshold *DT*. *GR* represents the horizontal distance between two neighboring particles in the simulated cloth to cover the terrain. As the *GR* decreases, the level of detail in the resulting digital terrain model becomes more refined. The *DT* determines whether the points are classified as ground or non-ground based on their distances from the cloth grid. Fewer ground points are obtained with a smaller *DT* value, while more points are separated as plants. We chose a grid resolution *GR* of 0.3 m and a distance threshold *DT* of 0.3 m as the parameter settings. The 0.3 m *GR* allowed for capturing sufficient details in the point cloud while maintaining computational efficiency. Similarly, the 0.3 m *DT* was suitable for accurately separating the ground and plant points, minimizing the likelihood of misclassification. The qualitative result can be seen in **Figure 3A**.

In the point cloud, the z-value of each point was the ellipsoidal height. Since we were interested in plant height which is the vertical distance between the ground level and the uppermost boundary of the primary photosynthetic tissues of a plant (excluding inflorescences) (Perez-Harguindeguy et al., 2016), the separated *PlantPCl* was normalized in height by subtracting the ground point elevation from all plant points to estimate plant height. By substituting the z-value of each plant point with the computed height difference, the height-normalized *PlantPCl* was obtained (**Figure 3B**).

#### 2.2.2 Row segmentation

**Figure 4:**
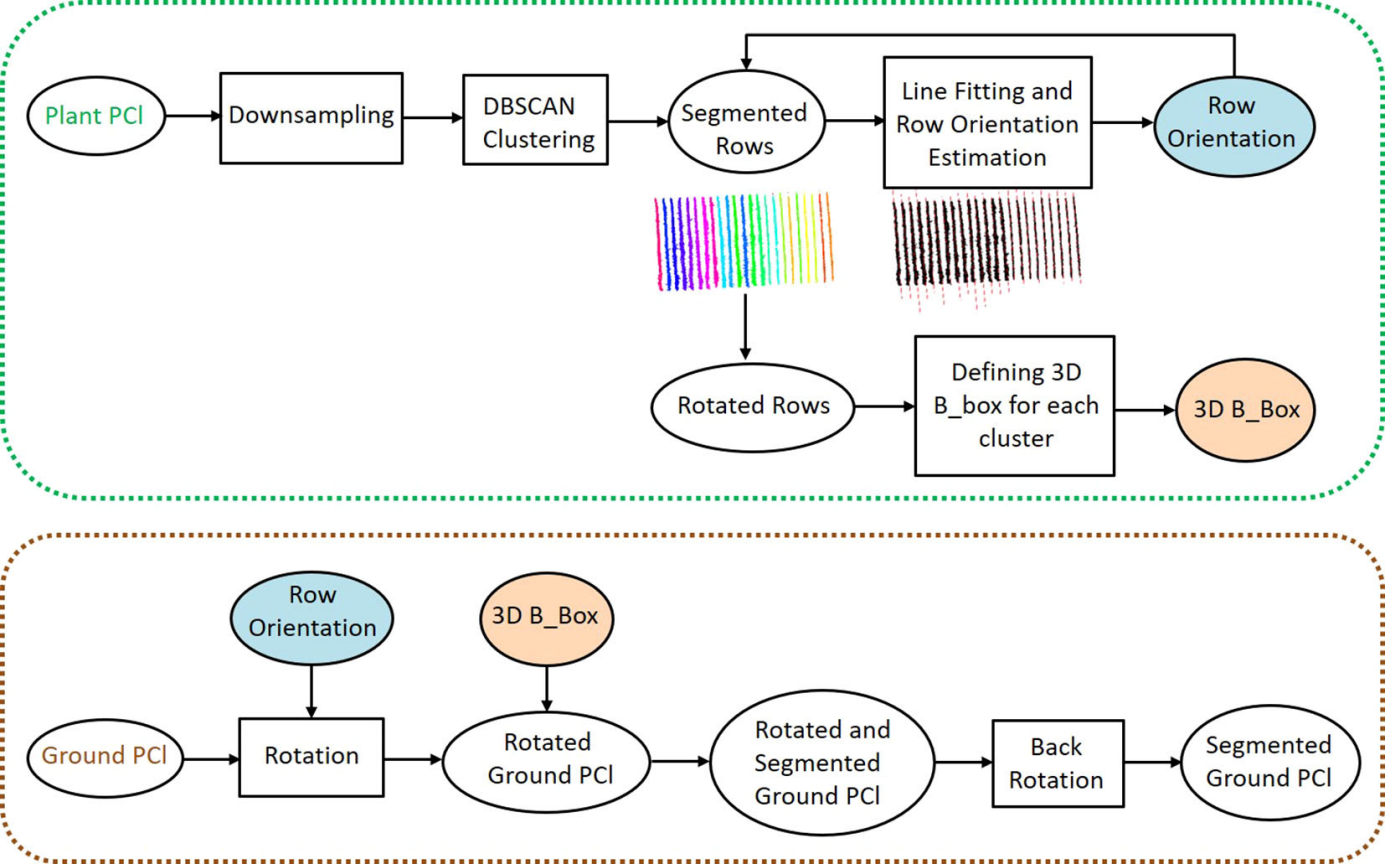
The proposed pipeline for the individual row segmentation.

We segmented the *PlantPCl* and *GroundPCl* into individual rows to extract plant parameters row-wise. Our pipeline for row segmentation consisted of two steps: segmentation of the *PlantPCl* into rows and defining a bounding box in 3D space to represent the spatial location of each segmented row to effectively segment the *GroundPCl* into rows as well. The flowchart of the proposed method for row segmentation can be seen in **Figure 4**.

First, we downsampled the *PlantPCl* to reduce the computational complexity and processing time of the algorithm using a 3D grid box with the size of (0.1 x 0.1 x 0.1 m). Second, we reduced the dimension to 2D by removing the z component. Assuming that the single rows do not overlap, we then applied the Density-based Spatial Clustering of Applications with Noise (DBSCAN) algorithm in the xy-plane. This algorithm was proposed by Ester et al. (1996) and is a density-based clustering algorithm intended to find clusters of any shape. DBSCAN relies on two main parameters: *ϵ*, the radius distance for point neighbors (Euclidean distance in our case), and *P_min_*, the minimum points needed to form a cluster (Amiruzzaman et al., 2022). We selected the parameters of the DBSCAN algorithm empirically based on the characteristics of the point cloud and the desired clustering outcome. After performing some preliminary experiments, we empirically set *ϵ* to 0.35 m, corresponding to the average inter-point distance in the downsampled *PlantPCl*, capturing closely located point clusters effectively. *P_min_* was set to 40 to identify clusters with a sufficient number of points while excluding noise, based on the point density distribution in the point cloud. This value was chosen based on the point density distribution in the downsampled point cloud, making it independent of the input point cloud density.

**Figure 5:**
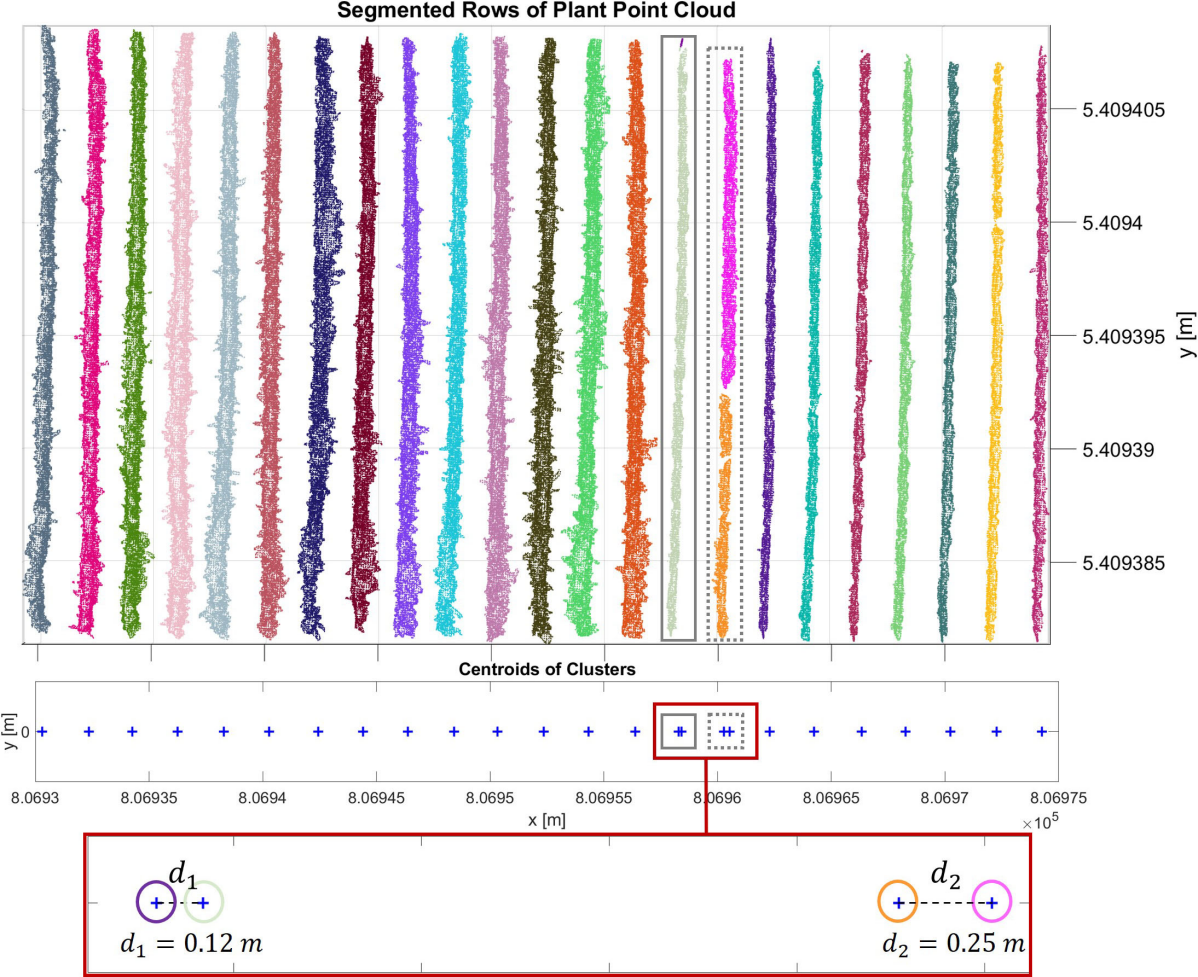
Initial result of the DBSCAN algorithm. Each row cluster is shown with a different color. Each centroid of the cluster is represented with a blue cross. In the grey rectangle (left), a whole row and a metal post in the row are clustered separately and are colored greenish-grey and purple, respectively. In the grey dashed rectangle (right), the row is segmented into two clusters due to the gap in the canopy. The clusters are shown in pink and orange colors. These centroids are analyzed more closely in the red frame. The distance between these pairs in grey and grey dashed rectangles respectively is less than the threshold value, therefore they are merged into the same cluster.

The initial result of the DBSCAN algorithm can be seen in **Figure 5**. Each row cluster is shown with a different color. However, these clusters may not be consistent due to gaps or missing plants. To address this issue, we improved the results by incorporating cluster centroids and leveraging the assumption that rows are linear. To achieve this, we fit a 2D line to the row clusters, allowing us to estimate the row orientation. Then, we rotated the point cloud with the row orientation angle *θ* parallel to the y-axis. After rotation, we calculated the centroids of the clusters and determined their similarity based on Euclidean distances *d* along the x-axis (**Figure 5**). Clusters with closely spaced centroids were merged into the same row, while clusters with larger centroid distances were considered separate rows. In our refinement method for row segmentation, we set the *d* between the centroids of clusters as 1 m by analyzing the datasets to address the issue of disjoint rows merging.

To segment the *GroundPCl* into rows, we used 3D bounding boxes that represent the spatial location of each segmented plant row. As explained before, since the rows had an orientation, it became challenging to compute the skewed boundaries of the bounding boxes. Therefore, we defined the 3D bounding boxes after obtaining the rotated rows. We used the maximum boundaries of the *PlantPCl* in the x and y direction and the boundaries of the *GroundPCl* for the z-direction to calculate the boundaries of the bounding boxes. To segment the *GroundPCl* into rows, we rotated it using the angle *θ* and used 3D bounding boxes for clustering.

#### 2.2.3 Row parameters extraction

##### 2.2.3.1 Trunk position

**Figure 6:**
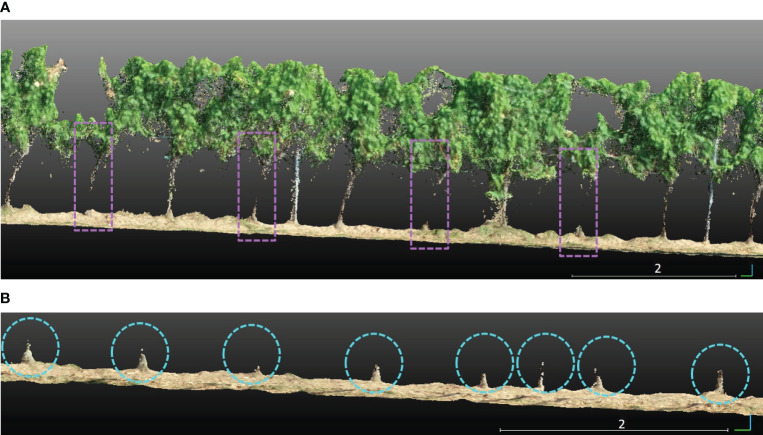
**(A)** Trunks that are not reconstructed well in the segmented plant row are shown in purple rectangles. **(B)** The trunks in a segmented ground row are encircled with cyan circles.

Identifying trunks under the canopy in vineyards with complex geometric structures is challenging. In our investigation in the *PlantPCl*, we observed that the thin structure of the majority of trunks was hard to reconstruct accurately due to the occlusions caused by the leaves, as can be seen in **Figure 6A**. On the other hand, the bottom part of the trunks was reconstructed better in the *GroundPCl* (**Figure 6B**). Furthermore, **Figure 6B** shows that the ground approximates a planar surface; in contrast, the trunks were observed with a different geometry clearly distinguished from the ground. We used geometric features to detect trunks in the *GroundPCl* to address this issue.

We proposed a pipeline for trunk detection in vineyards, leveraging features derived from the local neighborhood. To enhance computational efficiency, reduce noise, and improve feature stability, we downsampled the segmented row of the *GroundPCl* using a 3D grid box with dimensions of (0.05 x 0.05 x 0.05 m). This downsampling ensured a manageable density of points for subsequent calculations. Feature calculation involved two steps: computing the covariance matrix for the points within a local neighborhood around each point, defined by a specified radius *R* to analyze the variability of the point cloud in different directions, and determining eigenvalues of the matrix that provides insight into the principal axes of variability. The corresponding eigenvalues were sorted as *λ*_1_ ≥ *λ*_2_ ≥ *λ*_3_ ≥ 0. After conducting several experiments, we determined that the sphericity feature *S_λ_* = *λ*_3_ */λ*_1_ outperformed other features for trunk identification in the *GroundPCl*. For the sphericity feature calculation, we set the radius *R* to 0.25 m for the nearest neighbor search, as it effectively captured the approximate trunk diameter.

**Figure 7:**
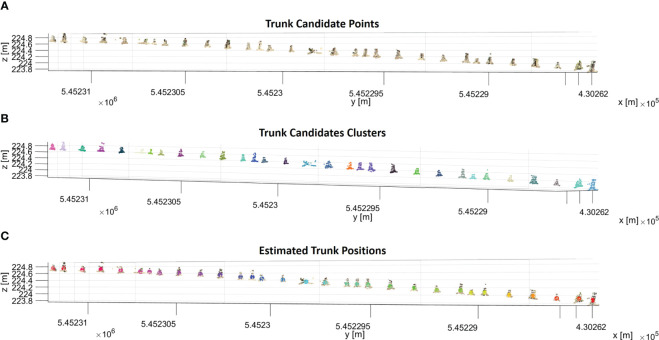
**(A)** Trunk candidate points that have a sphericity value larger than the sphericity threshold in a segmented ground row. **(B)** Trunk candidates are segmented into clusters with the DBSCAN algorithm, and each cluster of trunk candidates is shown in a different color. **(C)** The calculated centroid of each cluster is shown in different colors with trunk candidates.

We performed three steps to identify trunk candidates and estimate their 3D positions. Firstly, the sphericity values of each point in the segmented ground row were sorted into a histogram. We determined a threshold that helps us identify trunk candidates using Otsu’s method (Otsu, 1979). These candidates were the points with sphericity values exceeding the threshold (**Figure 7A**). Since trunk candidates stored many points for each trunk, the points belonging to the same trunk must be clustered. To achieve this, the DBSCAN algorithm was used to cluster the trunk candidates. The chosen parameters for the DBSCAN were 0.10 m and 5 for *ϵ* and *P_min_*, respectively. These parameter choices aided in effectively grouping the trunk candidates into meaningful clusters. **Figure 7B** illustrates the trunk candidates clusters, each represented by a distinct color. Finally, to estimate the position of each trunk, we calculated the 3D centroid of each cluster. By following these steps, we effectively identified trunk candidates and estimated their respective 3D positions in each segmented ground row, enabling a comprehensive analysis of the trunks in the given dataset (**Figure 7C**).

##### 2.2.3.2 Canopy characteristics

**Figure 8:**
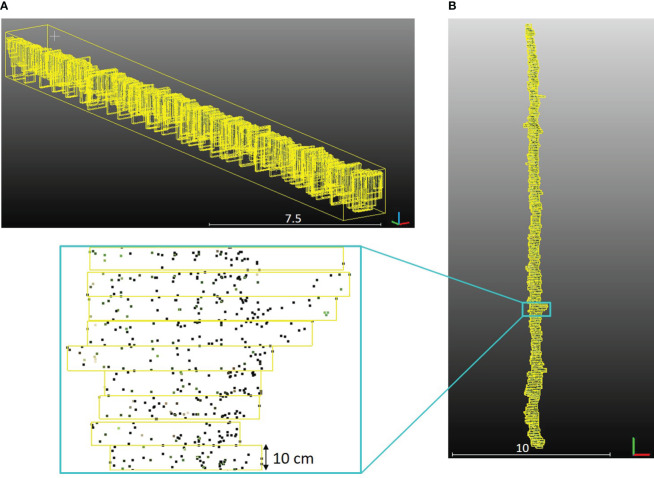
**(A)** Segments of the one row in the *PlantPCl* that were enclosed with a yellow bounding box in 3D space. **(B)** Top view of the segments along the row.

Canopy geometric parameters were extracted for each row of the *PlantPCl* with a high spatial resolution along the row. Our approach involved segmenting each row into 3D bounding boxes of equal length along the y-axis according to the methodology described in Escolà et al. (2017); Cabrera-Pérez et al. (2023), and Escolà et al. (2023). Segments, represented by yellow bounding boxes, were visually depicted along a single row in **Figure 8A**. Furthermore, a top and close-up view of the segments was presented in **Figure 8B**. Each parameter was computed in each segment along the row. By doing this, we can achieve a detailed analysis of parameters along a row by adjusting the number of segments. Therefore, any desired resolution of the parameter estimation along the row can be achieved. To ensure a high level of detail, we chose the number of segments to be 250. This decision resulted in a consistent segment length of 10 cm along the row. Comprehensive information and visualizations regarding each plant parameter along each row were provided through detailed diagrams and histograms. In the following, we described the parameters extracted by our pipeline.

**Plant Height:** The shortest distance from the ground to the highest point of the canopy in the z-axis is defined as plant height (Perez-Harguindeguy et al., 2016). We used a method based on the 90th percentile of normalized heights, as shown in a prior study (Becirevic et al., 2019), which highlighted the reliability of the UAV-based crop height extraction. We also visually inspected the data to ensure our choice of percentile was appropriate. By calculating the mean of the z-values within the 90th percentile of the normalized height, we obtained height estimations for each segment.

**Canopy Width:** Assuming that each row in the *PlantPCl* was positioned on the xy-plane and aligned parallel to the y-axis following the rotation and ground projection, we can define the canopy width as a distance perpendicular to the y-axis. In our pipeline, we calculated the canopy width as the difference between the mean of the y-values within the 90th percentile and the mean of the y-values within the 10th percentile, following the procedure already described for the plant height. In this way, we obtained a more accurate estimation of the canopy width while excluding extremes.

**Figure 9:**
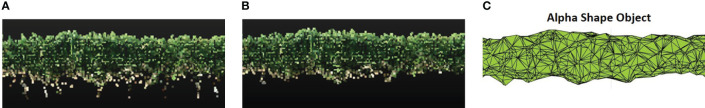
**(A)** Side view of the segmented row in *PlantPCl*. **(B)** Filtered point cloud. Trunk and branch pieces are removed with the SOR filter. **(C)** The alpha shape object of the row.

**Canopy Volume:** The canopy volume is a reliable indicator of the overall health and vigor of plants (Arnó et al., 2013; Caruso et al., 2017; Escolà et al., 2017). The estimated vine canopy volume becomes particularly valuable in assessing vigor, especially where a single measure, such as height and width, is insufficient to understand canopy geometry. To calculate the canopy volume we employed the alpha shape algorithm (Edelsbrunner et al., 1983), which generates a bounding volume encompassing a set of plant points. However, to accurately calculate the canopy volume, it was necessary to discard pieces of trunks or single branches that were occasionally present in the *PlantPCl*, as illustrated in **Figure 9A**. As explained before, since the trunks were not reconstructed well, the number of trunk points was significantly lower than the canopy points in the *PlantPCl*. To address this, we applied the Statistical Outlier Removal (SOR) filter to eliminate these points within the row. As a result, we obtained a filtered *PlantPCl* that excludes trunk or branch pieces, as illustrated in **Figure 9B**. After filtering, the alpha shape approach was employed with different alpha radius *α*. The parameter *α* is the sphere’s radius that sweeps over the points to create the alpha shape and is used to tighten or loosen the object. The approach, in theory, uses an optimal alpha value to approximate bounding volume; however, finding an optimal value is extremely difficult (Yan et al., 2019). Therefore, we empirically chose an alpha radius representing the canopy’s concave structures without creating disconnected objects. Through empirical observations in our study, we determined the value of *α* as 0.3 m for the alpha shape object when calculating the canopy volume (**Figure 9C**).

**Canopy Lower Bound:** The filtered *PlantPCl*, which excludes the trunks, holds significant importance in facilitating a comprehensive analysis of the canopy structure. In the filtered *PlantPCl*, we can define the canopy lower bound as the lowest point that belongs to the canopy on the z-axis. In our pipeline, the canopy lower bound was estimated as the mean of the z-values within the 10th percentile. Thus, by incorporating this canopy characteristic, we obtain valuable insights into the vertical structure of the canopy, enabling a more detailed examination of the vegetation distribution and its variability along the row.

### 2.3 Evaluation method

#### 2.3.1 Row segmentation

To evaluate the accuracy of the segmented plant rows, the point clouds of the segmented rows were compared with the manually segmented rows. The evaluation involved comparing the number of points in the segmented row with the number of points in the corresponding manually segmented row. Segmentation accuracy was calculated for each row considering the overlap between the segmented row and the ground truth one. Furthermore, we took into account the training system associated with each row. This analysis provided insights into the performance of the segmentation method across different training systems.

#### 2.3.2 Trunk position

Our study focused on accurately estimating the positions of individual trunks within vineyard rows. We evaluated the accuracy of our estimations by comparing them to ground truth data which is manually selected trunks in the point cloud. To determine the accuracy of our estimates, we defined a 15 cm search radius and checked if any ground truth trunks were within this radius. Then the confusion matrix was calculated based on the presence or absence of ground truth trunks. The evaluation of our estimated trunk positions involved calculating true positives (TP), false positives (FP), and false negatives (FN). We computed precision, recall, and F1 scores for each dataset to assess the performance of trunk detection. Precision focuses on the quality of the detected trunks, measuring the extent to which the identified trunks are valid. On the other hand, recall, in our case, quantifies the capability to accurately identify actual trunks, emphasizing the detection rate. To obtain a comprehensive evaluation, we utilized the F1 score, which offers a balanced assessment of the overall performance, considering both recall and precision.

Furthermore, we conducted an additional experiment to investigate the impact of different flight parameters on the trunk detection method. To do so, we applied our method to three distinct datasets, *tilted_20m*, *nadir_20m*, and *nadir_15m*, and subsequently compared the obtained results.

#### 2.3.3 Canopy characteristics

In previous studies (De Castro et al., 2018; Di Gennaro and Matese, 2020; Mesas-Carrascosa et al., 2020), UAV-based canopy geometric parameter estimation has demonstrated higher accuracy compared to ground truth measurements; however, the validity of such comparisons is highly dependent on the specific sample positions, rendering them less meaningful. For instance, ground truth volume measurements are highly subjective and depend on the specific person taking the measure (Colaço et al., 2017; Qi et al., 2021). These measurements are also time-consuming, with the additional limitation of not accounting for canopy gaps. Furthermore, certain studies have indicated that manual measurements tend to overestimate canopy thickness by approximately 30% when compared to LiDAR-based measurements (Gil et al., 2014). Therefore, considering these challenges, we rely on the proven reliability of parameter estimation methods, as demonstrated in previous research, and do not directly compare our estimates with reference measures.

## 3 Results and discussion

### 3.1 Row segmentation

**Figure 10:**
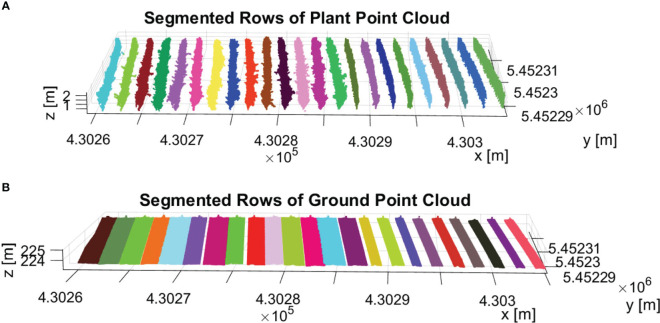
Result of row segmentation. The rows are color-coded, with each row represented by a different color. **(A)** Segmented rows of *PlantPCl*. **(B)** Segmented rows of *GroundPCl*.

The final result of row segmentation is visually represented in **Figure 10**. **Figure 10A** illustrates the segmentation results for the *PlantPCl*. The rows are color-coded, with each row represented by a different color. Our segmentation method accurately separates the *PlantPCl* into distinct rows, therefore we can have a clear understanding of the spatial distribution and arrangement of the plants within the vineyard. By employing our proposed method, we successfully address the challenges posed by gaps or missing plants in the canopy, resulting in accurate and consistent row segmentation. Similarly, **Figure 10B** shows the segmentation result for the *GroundPCl*. The ground points are assigned different colors based on the rows they belong to. This segmentation allows for a comprehensive analysis of the ground characteristics along each row, which is interesting for individual trunk detection.

As a result, the row segmentation method demonstrated outstanding accuracy in all datasets, achieving a flawless segmentation rate of 100%, in line with existing research (Jurado et al., 2020; Moreno and Andújar, 2023). Notably, our analysis revealed that the training systems had no noticeable effect on the performance of the row segmentation method. Furthermore, the comparison of different flight parameters revealed no impact on the accuracy of row segmentation.

### 3.2 Row parameters extraction

#### 3.2.1 Trunk position

**Figure 11:**
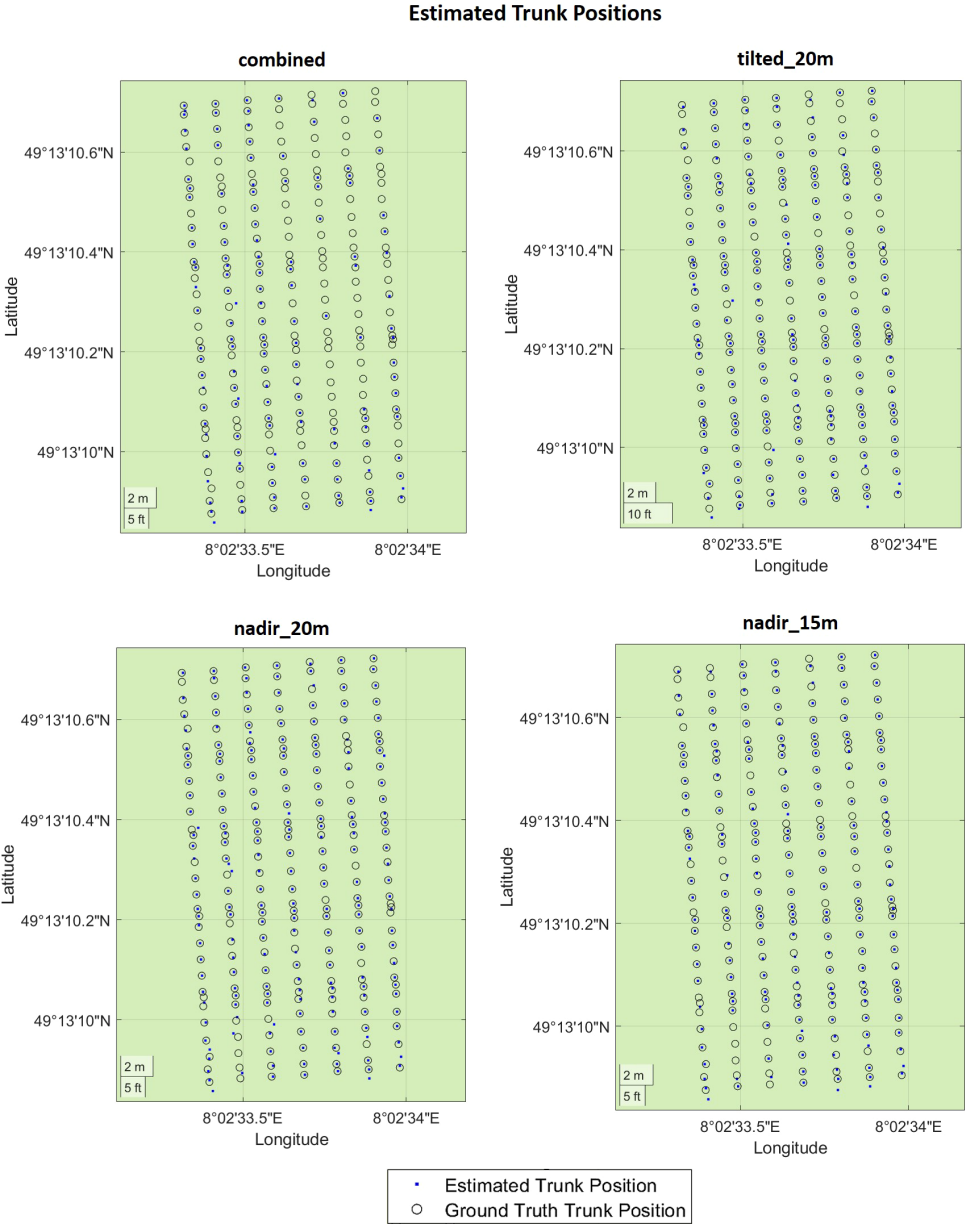
Evaluation of the trunk positions in geographic coordinates. The estimated trunk positions are colored in blue. The black circles represent the ground truth trunk positions.

We presented the estimated trunk positions and ground truth data in **Figure 11**, where blue dots represent estimated trunks and black circles represent ground truth trunks. **Table 2** shows the corresponding numerical evaluation. In *combined* dataset, trunk positions in the SMPH training system were estimated with a higher F1 score of 76% compared to 59% F1 score in the VSP training system. It can be seen in **Figure 11** that trunks were not detected well in rows number 4, 5, and 6. This is because, in this specific dataset, these trunks belonging to these rows were not properly reconstructed by the SfM pipeline due to the occlusions caused by the canopy.

**Table 2:** Evaluation of detected trunks.

	Training system	TP	FP	FN	Precision	Recall	F1 score
*combined*	SMPH	66	14	27	83%	71%	76%
	VSP	54	5	70	92%	44%	59%
*tilted_20m*	SMPH	83	9	10	90%	89%	90%
	VSP	101	10	23	91%	81%	86%
*nadir_20m*	SMPH	73	19	20	79%	78%	79%
	VSP	111	11	13	91%	90%	91%
*nadir_15m*	SMPH	67	11	26	86%	72%	78%
	VSP	109	12	15	90%	88%	89%

The evaluation revealed that the highest F1 score of 91% was achieved in *nadir_20m* dataset with the VSP training system. This is because, in the VSP case, the canopy volume is much lower so that a larger portion of the trunk is visible from the camera, which results in a higher precision on all datasets for our pipeline. Considering both training systems, the highest F1 score was achieved with the *tilted_20m* dataset, indicating that the trunks were detected better in this dataset. Specifically, we observed that the more inclined camera angle outperformed the nadir angle in effectively detecting plants under the canopy. This confirms existing research on both maize (Che et al., 2020) and grapevines (García-Fernández et al., 2021) where a more inclined camera angle was more effective for the point cloud reconstruction. However, in existing works, no assessment of the trunk detection performances was investigated.

The lowest F1 score of 59% was achieved in the *combined* dataset. Although being reconstructed using images captured from different camera angles and flight heights, the *combined* dataset did not yield an improvement in performance. Interestingly, the individual datasets displayed significantly higher recall rates when compared to the *combined* dataset. These findings suggest that the variability in data introduced by the different flight parameters did not enhance the overall detection outcome. Based on these results, it is evident that selecting a specific flight parameter set, rather than combining data captured using different flight parameters, provided superior trunk detection performances.

We can see from the results that there is no substantial difference in terms of F1 score between the *nadir_20m* and *nadir_15m* datasets. These datasets were both captured from a nadir angle but at varying flight heights, enabling us to assess the influence of flight height on plant detection directly. Notably, we discovered that higher flight height can still yield accurate results with lower data density but a larger field of view, potentially shortening data collection time.

Although existing research achieved high accuracy for grapevine detection, some conditions were assumed regarding either plant distributions (Milella et al., 2019; Pádua et al., 2020) or the absence of the canopy (Jurado et al., 2020; Di Gennaro et al., 2023). Instead, in our work, we assess the trunk detection performances without any assumptions about the vineyard conditions. In fact, we tested our pipeline with different training systems, irregular plant spacing, and the presence of a fully developed canopy, achieving a precision of 92%. It is important to note that, although in our case most plants within the vineyard were effectively reconstructed, the thin-structured trunks located under the canopy posed a challenging scenario for accurate trunk detection. Our research findings highlight the impact of camera angle and flight height when designing aerial imaging surveys for plant detection within vineyards. By optimizing these parameters, researchers and practitioners can substantially improve performances in individual trunk identification.

#### 3.2.2 Canopy characteristics

**Figure 12:**
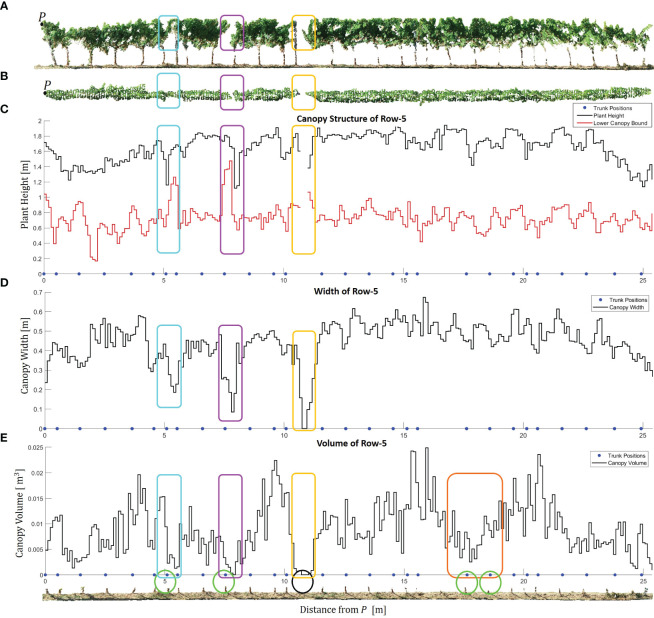
Canopy characteristics of Row-5. **(A)** Side view of the row. Point *P* marks the starting point of the row, representing the location with the minimum y-coordinate. The cyan, magenta, yellow, and orange rectangles highlight significant regions of interest where the canopy geometry changes abruptly. **(B)** Top view of the row. **(C)** Estimated plant height and lower canopy bound along the row. The blue points indicate the estimated positions of the trunks. **(D)** Estimated canopy width along the row. **(E)** Estimated canopy volume along the row. The circles within the *GroundPCl* of Row-5 indicate the positions of the estimated trunks related to abrupt changes in the canopy.

**Figure 13:**
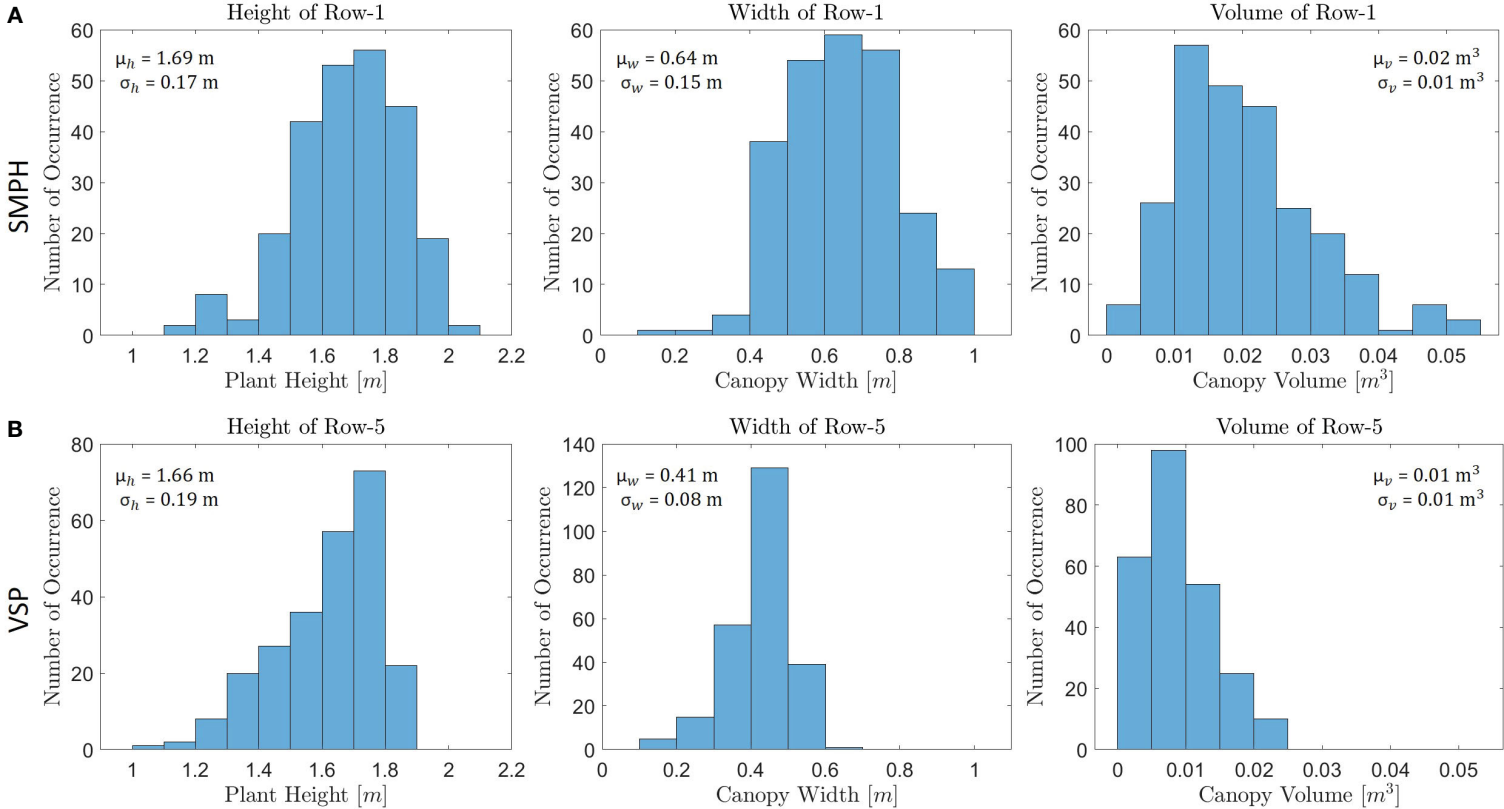
A comparison was conducted between **(A)** Row-1 in the SMPH training system and **(B)** Row-5 in the VSP training system in terms of their training systems, using histograms to analyze the row parameters.

In **Figures 12** and **13**, we show the distribution of the canopy characteristics along a subset of rows using our segment-based approach. **Figure 12** provides valuable and comprehensive insights into the canopy characteristics and trunk positions in Row-5 trained in the VSP system. The side view and top view of the row are shown in **Figures 12A, B**, respectively. It allows us to observe the changes in canopy geometry along the row, beginning from point *P* to the end of the row, with the highlighted regions of interest indicating areas where the canopy structure undergoes abrupt variations.

By analyzing the estimated plant height and the lower canopy bound in **Figure 12C**, we gain a deeper understanding of the vertical structure of the plants. The blue points representing the estimated trunk positions allowed us to relate the plant positions to the respective canopy structures. The proposed method for height estimation provided us with not only height estimation but also identification of the missing plants along the row. We observed an interruption and sharp fall in the graph in the absence of plants as can be seen in yellow rectangles. The absence of the related trunk in the black circle in *GroundPCl* proved our analysis as can be seen in **Figure 12E**. Furthermore, the estimated canopy width in **Figure 12D** revealed variations in the lateral extent of the canopy along Row-5. In particular, we observed sudden declines in the graph, highlighted with magenta, cyan, and yellow frames in **Figure 12D**. It is clear that the canopy width has decreased in these frames, as can be seen in **Figure 12B**.

The graph of the estimated canopy volume in **Figure 12E** provided a quantitative measure of the three-dimensional extent of the canopy, reflecting the overall vegetative vigor and biomass accumulation. This specific alpha radius selection allowed for accurately representing concave structures within the canopy while avoiding creating disconnected or fragmented objects. While no significant change was observed in plant height and width, there was a significant decrease in canopy volume as can be seen in the orange frame. Therefore, when combined with plant height and canopy width, canopy volume provided a holistic perspective on the vineyard’s canopy architecture, allowing for a more accurate assessment of its health and growth dynamics (Escolà et al., 2017).

We further focused on the comparison between the results for two training systems, VSP and SMPH. **Figure 13** shows the histogram of row parameters for two exemplary rows that are Row-1 (trained in the SMPH system) and Row-5 (trained in the VSP system). The expected difference in plant height, canopy width, and volume explained in Section 2.1 could be precisely detected between the two training systems. As shown in **Figure 13**, the mean height, width, and volume of Row-1 are much larger than Row-5, as in the SMPH system the canopy volume is typically denser than in VSP. Although we show histograms just for two rows, we also computed the mean of the row parameters trained with the SMPH and VSP system separately for each dataset. In this way, we investigated both the differences in row parameters for the two training systems and the influence of the different flight settings, as shown in **Table 3**. Similarly to the Row-1/Row-5 results, we observed that the mean plant height, canopy width, and canopy volume of the rows trained in the SMPH system were higher than those trained in the VSP system for all datasets. Our results found no significant differences in plant height and canopy width across *tilted_20m*, *nadir_20m*, and *nadir_15m* datasets in two training systems. However, a relatively lower canopy volume was observed in the SMPH training system within the *nadir_15m* dataset. The reason for this may be the lower flight altitudes can yield a higher spatial resolution, which makes the system more sensitive to small canopy variations potentially causing an underestimation of canopy volume.

**Table 3:** Row parameters in different datasets.

Parameters:	Plant height [*m*]				Canopy width [*m*]				Canopy volume [*m* ^3^]			
	SMPH		VSP		SMPH		VSP		SMPH		VSP	
Training system:	Mean	SD	Mean	SD	Mean	SD	Mean	SD	Mean	SD	Mean	SD
*combined*	1.72	0.04	1.66	0.03	0.66	0.03	0.35	0.05	0.61	0.03	0.23	0.02
*tilted_20m*	1.71	0.03	1.61	0.02	0.69	0.03	0.36	0.04	0.59	0.03	0.24	0.02
*nadir_20m*	1.70	0.06	1.58	0.01	0.70	0.03	0.36	0.05	0.55	0.02	0.23	0.02
*nadir_15m*	1.71	0.04	1.58	0.01	0.71	0.03	0.37	0.05	0.46	0.02	0.21	0.01

One of the notable advantages of our method is its flexibility in achieving the desired resolution of parameter calculations. By adjusting the number of segments, we can readily modify the resolution along the row. Compared to other methods that fix the segment length *a priori* (Escolà et al., 2017; Cabrera-Pérez et al., 2023), we can precisely analyze and evaluate the plant’s characteristics and variations along the row at the desired level of detail. One example of this is given in **Table 3**, where the canopy volume is computed with a segment length of 1 m instead of 10 cm to give a more reasonable value. Overall, our approach provides a robust and adaptable framework for obtaining essential plant parameters, offering valuable insights into the spatial distribution and properties of the plant along the row.

### 3.3 Algorithm application on LiDAR dataset

**Figure 14:**
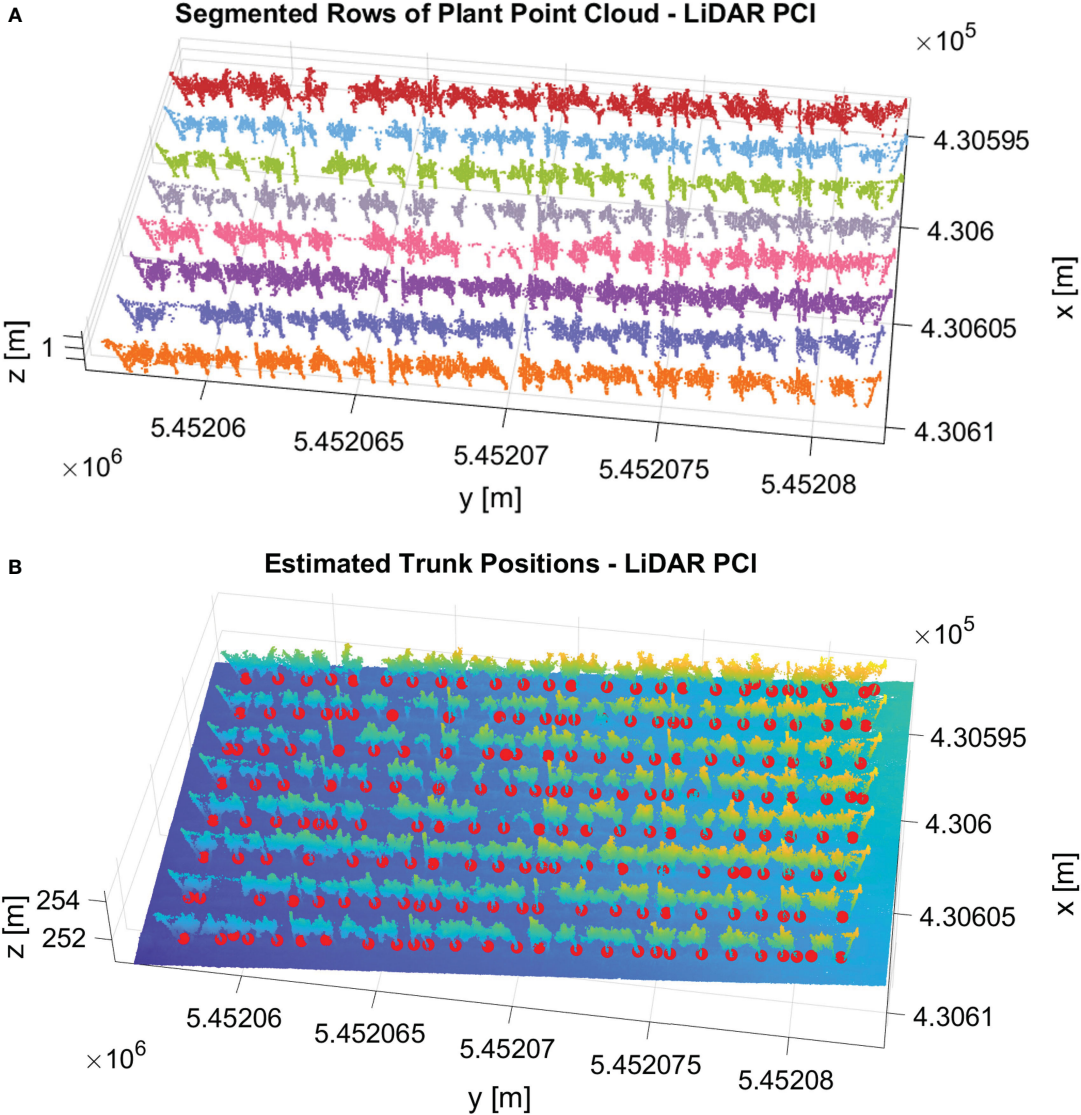
Results of our pipeline in the vineyard’s LiDAR point cloud dataset. **(A)** The row segmentation output. Each segmented row is represented with a different color. **(B)** The result of the trunk detection algorithm. The detected trunks are shown with red spheres.

We successfully applied our pipeline to the vineyard’s LiDAR point cloud dataset. **Figure 14** shows the qualitative results of row segmentation and trunk detection algorithm. We utilized the same set of parameters used in the image-based datasets for all components within the pipeline. Remarkably, our method effectively segmented the point cloud into rows with 100% accuracy. **Figure 14A** shows the qualitative result of row segmentation. Notably, our trunk detection algorithm also succeeded in identifying trunks, achieving an F1 score of 77%. **Figure 14B** shows the detected trunks with red spheres. This experiment demonstrated that our algorithm can generalize effectively to different sensor settings with accurate results without parameter tuning.

## 4 Conclusion

The determination of the geometric properties and the identification of the individual plants were the main focus of this work, and the results presented demonstrate the potential of using a 3D plant model derived from RGB images acquired with a UAV for achieving these objectives in the vineyard. The extraction of single rows was performed. Consequently, for each row, we derived the plant positions as well as detailed row parameters, including the plant height, canopy width, and canopy volume. In contrast to many other approaches, we provided detailed information and visualizations of the height, width, and volume in the form of diagrams and histograms, giving essential clues on the distribution of these factors along the row. These extracted parameters have the potential to enhance vineyard productivity, improve grape quality, and contribute to the long-term sustainability of vineyard operations. This detailed geometric analysis of the canopy offers valuable insights for vineyard managers and breeders, assisting them in crucial tasks such as pruning, agrochemical spraying, and optimizing yields. Additionally, the influences of different flight parameters on the extracted plant parameters have been investigated. The whole pipeline is independent of the terrain slope and does not require assumptions like plant or row spacing. We investigated all these parameters in detail and had reference data for the segmented rows and estimated trunk positions for the evaluation. The vine rows were segmented with a high accuracy of 100% in the vineyard plot independent of the training systems and different flight parameter settings. We could also identify the trunk positions with a precision of 92%. Furthermore, we applied our algorithm to the LiDAR point cloud and showed accurate results regarding row segmentation and trunk detection. This experiment demonstrates that our algorithm can generalize to different sensor settings with good performances without the need for parameter tuning.

## Data availability statement

The raw data supporting the conclusions of this article will be made available by the authors, without undue reservation.

## Author contributions

MC designed and conducted the analyses. LK and HK helped to initiate the work. The development of the method was done by MC, guided by LZ and LK. LK helped to co-design the experiments. DP and AD collected and preprocessed the data. LZ contributed to the data preparation. MC, LZ, and LK contributed to the writing of the manuscript. All authors contributed to the article and approved the submitted version.
